# Evaluation of safety and immunogenicity of duck-plague virus gC/gE double gene deletion

**DOI:** 10.3389/fimmu.2022.963009

**Published:** 2022-08-18

**Authors:** Peilin Ruan, Xin Feng, Anchun Cheng, Mingshu Wang, Wei Zhang, Ying Wu, Qiao Yang, Bin Tian, Xuming Ou, Di Sun, Shaqiu Zhang, Sai Mao, Dekang Zhu, Renyong Jia, Shun Chen, Mafeng Liu, Xin-Xin Zhao, Juan Huang, Qun Gao, Yanling Yu, Ling Zhang, Leichang Pan

**Affiliations:** ^1^ Institute of Preventive Veterinary Medicine, Sichuan Agricultural University, Chengdu, China; ^2^ Key Laboratory of Animal Disease and Human Health of Sichuan Province, Sichuan Agricultural University, Chengdu, China; ^3^ Avian Disease Research Center, College of Veterinary Medicine, Sichuan Agricultural University, Chengdu, China; ^4^ R & D Department, Sinopharm Yangzhou VAC Biological Engineering Co., Ltd., Yangzhou, China

**Keywords:** duck plague, gC, gE, gene deletion, immunogenicity, vaccine

## Abstract

Duck plague caused by duck plague virus (DPV) is a highly contagious disease that can cause serious morbidity and death in waterfowl such as ducks and geese, and bring huge economic losses to the duck industry. In this study, on the basis of the duck plague virus gC gene deletion strain CHv-ΔgC, based on the duck plague virus bacterial artificial chromosome (BAC) platform in our laboratory, the gE gene was knocked out using the traceless deletion technology to obtain gC/gE double gene deletion candidate vaccine strain CHv-ΔgC/gE. The double gene deletion strain (CHv-ΔgC/gE) constructed in this study has greatly weakened virulence, no pathogenicity to ducks, and stable genetic characteristics *in vitro* and *in vivo*. Ducks immunized with CHv-ΔgC/gE can produce neutralizing antibodies and ELISA antibody levels comparable to those of commercial duck plague attenuated vaccine immunization, and can resist 100 LD_50_ CHv challenge of ducks, with good immune protection effect. It has the potential to be further developed into duck plague gC/gE double gene deletion, marked attenuated vaccine.

## Introduction

Duck plague (DP), also known as duck viral enteritis, is caused by Duck Plague Virus (DPV), which belongs to the *Alphaherpesvirinae* and *Mardivirus* genus of the *Herpesviridae* family (1). The structure of DPV virus from outside to inside is envelope, tegument, capsid and core, capsid is icosahedral, genome is linear double-stranded DNA, DPV genome consists of unique long region (UL), intermediate repeat sequence (IRS), unique short region (US) and terminal repeat sequence (TRS), including 78 open reading frames (ORFs), of which 65 ORFs are located in the UL region, 11 ORFs are located in the US region, and the remaining two ORFs are located in the IRS region and TRS region (1–6). DPV is a pan-tropic virus that can replicate and proliferate in various tissues and organs of the body, epithelial cells and lymphocytes are its main replication sites, the digestive tract and immune organs of ducks are most severely damaged after DPV infection (7–10). At the beginning of infection, DPV can establish latent infection in the trigeminal ganglion (TG), and DPV may be reactivated when the host immunity is low, resulting in the recurrence of duck plague (11). The typical clinical symptoms of duck plague are high fever, increased eyelid secretions, edema of the head and neck, and discharge of green or grayish-white loose stools. Ducks generally die within 1-5 days of clinical symptoms (8, 12, 13).

Vaccination is one of the most effective ways to control the disease and reduce economic losses in the duck industry. Since the inactivated vaccine does not have the ability to replicate in animals, it mainly causes the body to produce humoral immunity, and the induced immune response lasts for a short time, requiring multiple immunizations to achieve long-term protection. It cannot adapt to our country’s new goal of eradication duck plague, so the use of inactivated vaccines is gradually decreasing. The live attenuated vaccines currently on the market are mainly chicken embryo attenuated duck plague virus and double attenuated vaccine with duck hepatitis virus (14). However, the genetic background mechanism of the weakened duck plague virus is unclear, and the immunized ducks cannot be distinguished from ducks naturally infected with duck plague virus, which is not conducive to the elimination of infected ducks and the eradication of duck plague.

The gC and gE of DPV are encoded by the UL44 gene and the US8 gene respectively. They are the main components of the envelope and belong to multifunctional transmembrane proteins. In alphaherpesviruses, the gC gene is a non-essential gene for viral replication, but gC is the main adsorption protein, which plays an important role in the process of viral adsorption and entry into host cells (15–19), and its deletion can affect the virulence of the virus. The extracellular and intracellular domains of gE proteins have different functions, among which the extracellular domain mainly affects the cell-to-cell transmission of virions on epithelial and neural tissues (20). The ability of the virus to transmit from nerve cells to epithelial cells was lost after mutation of the gE ectodomain, and the virus could not be transmitted from epithelial cells to nerve cells in the absence of gE (21, 22). In this study, we constructed a double gene deletion strain CHv-ΔgC/gE based on the *gC* deletion strain CHv-ΔgC, and evaluated its pathogenicity and immunogenicity in ducklings. The results showed that compared with the commercial duck plague attenuated vaccine, CHv-ΔgC/gE had the same level of protection efficacy and safety. Therefore, it is a promising candidate vaccine for the prevention and control of duck plague.

## Materials and methods

### Cells and viruses

Duck embryo fibroblasts (DEFs) were prepared from 9-day-old duck embryos and cultured in DMEM medium (Gibco, Shanghai, China) containing 10% neonatal bovine serum (NBS, Gibco, Rockford, USA) according to conventional methods (23). Duck plague virus Chinese virulent strain (CHv) (GenBank: NO. JQ647509.1) (5), gC deletion strain (DPV-CHv-ΔgC) and GS1783-BAC-CHv-ΔgC infectious clone strain, pEP- Kan-S plasmid was preserved and provided by the Poultry Disease Control Research Center of Sichuan Agricultural University.

### Generation and identification of CHv-ΔgC/gE

The CHv-ΔgC/gE double-gene deletion strain was obtained based on CHv-ΔgC using the DPV-BAC infectious cloning platform constructed in our laboratory through RED two-step homologous recombination, the method was referred to the previous study (24). Briefly, the Kan template was amplified with pEP-Kan-S, and the ΔgE-Kan targeting fragment was amplified by PCR using the gE targeting primer; the ΔgE-Kan fragment was homologously recombined into GS1783-ΔminiF bacteria containing the BAC-CHv-ΔgC genome. Next, the Kan gene was removed and the gene of interest was identified by PCR using gE identification primers and sequenced, while the infectious clones were subjected to RFLP analysis. The plasmid with the correct sequence was transfected into DEFs, and plaque-purified to obtain the virus CHv-ΔgC/gE, which was identified by IFA and WB with gE polyclonal antibody. The primer sequences have been described elsewhere (23, 25, 26) (**Table 1**).

**Table 1:** Primers of construction and identification of deleted recombinant virus.

Primer name	Sequence(5’-3’)	Purpose
ΔgE-Kan-F	ATACTGCCGGCCAGACTACGGAACC TCAACAATTGGTACGTAGGGATAAC AGGGTAATCGATTT	Replacement of the *gE* gene by the kan cassette
ΔgE-Kan-R	TAACTATTTCACTAGTGAGTCATTA GTTCAACATCCATGACGTACCAATT GTTGAGGTTCCGTAGTCTGGCCGGC AGTATGCCAGTGTTACAACCAAT	
gE-F	TCTCAAGACGCTCTGGAATC	Identification of the *gE* gene deletion
gE-R	AGCGAGTACTTCTCTGCGTC	
gC-F	GAAGGACGGAATGGTGGAAG	Identification of the *gC* gene deletion
gC-R	AGCGGGTAACGAGATCTAATATTGA	
gC probe	CCAATGCATCGATCATCCCGGAA	
UL30-F	TTTTCCTCCTCCTCGCTGAGT	Identification of the *UL30* gene deletion
UL30-R	GGCCGGGTTTGCAGAAGT	
UL30 probe	FAM-CCCTGGGTACAAGCG-MGB	

### Growth curve and genetic stability of CHv-ΔgC/gE

To determine the growth curve of CHv-ΔgC/gE, DEFs cultured in 12-well plates were infected with 0.01 MOI of CHv-ΔgC/gE, CHv-ΔgC and CHv, respectively, MEM containing 2%NBS was then added. All samples of cells and supernatants were collected at 24 h, 48 h, 72 h, 96 h, 120 h and 144 h after infection. Virus titers in samples were determined by determining the 50% tissue culture infectious dose (TCID_50_). The gene stability of CHv-ΔgC/gE was determined by PCR detection of gE deletion during *in vitro* (14-day-old Peking ducks) and *in vivo* (DEFs) passage.

### Challenge experiment in ducklings

For the pathogenicity experiment, 80 14-day-old ducklings were randomly divided into groups of 10 and injected with 1 mL of CHv-ΔgC/gE (10^4^-10^6^ TCID_50_/mL), CHv-ΔgC (10^4^-10^6^ TCID_50_/mL), CHv (100 LD_50_/mL) and MEM respectively, all were intramuscular injections in the right leg. The rectal temperature, mental state and survival of ducks in each group were recorded every day after challenge. The dead ducks were dissected immediately, and the surviving ducks in each group were killed after 10 days of infection. The visceral samples were collected to make microscopic pathological sections for pathological observation. The distribution of CHv-ΔgC/gE in ducks was detected by fluorescence quantitative PCR (25).

### Protective efficacy experiment in ducklings

In order to test the immunogenicity and the protective effect of immunized ducks against virulent challenge, 30 14-day-old ducklings were divided into 3 groups, the first group was inoculated with 1 mL CHv-ΔgC/gE (10^6^ TCID_50_/mL), the second group was inoculated with a dose (10^7.7^ copies/dose) of live duck plague vaccine CVCC AV1222 (AV1222, VAC, Yangzhou, China), the third group was the control group, inoculated with 1 mL of MEM. After 10 days of immunization, all ducks were challenged with 100 LD_50_ CHv, the methods of immunization and challenge were intramuscular injection in the right leg (23). Body temperature, body weight and survival were recorded daily after challenge.

### Serological tests

Serum samples were collected at different time points, and the DPV-UL55 protein-specific antibody levels in serum were detected according to the established ELISA method (27). The level of neutralizing antibodies in the immunized ducks was determined by the fixed virus-diluted serum method. Briefly, 0.1 mL of 10-fold diluted serum (heat inactivated at 56° C. for 30 minutes) was mixed with an equal volume of 200 TCID_50_/0.1mL of DPV, and neutralizing antibodies were detected by conventional methods (23). NAbs titers were expressed as the maximum dilution at which 50% of the virus was neutralized.

### Histopathology

The collected tissues were fixed with 4% paraformaldehyde fixative for 7 days, followed by tissue trimming, alcohol gradient dehydration, xylene clearing, and paraffin embedding, and then cut into 4 μm thick sections, and stained with hematoxylin and eosin (H&E), and examined by light microscopy, after mounting with neutral resin.

### Real-time PCR

Using the quantitative primers in **Table 1**, the viral load in the tissue and organ samples of each infected animal was detected by real-time PCR method (25). Gene copy numbers for each sample were expressed as log_10_ copies per 0.1 g or 0.1 mL of tissue sample.

### Statistical analysis

All data were subjected to Student’s t-test with GraphPad Prism 5.0. Results are presented as mean ± standard deviation (SD), p values are indicated by asterisks, ns means not significant (p > 0.05); * means p < 0.05, ** means p < 0.01, ** means p < 0.001, p < 0.05 was considered significant.

## Results

### Construction and identification of deletion virus CHv-ΔgC/gE

**Figure 1:**
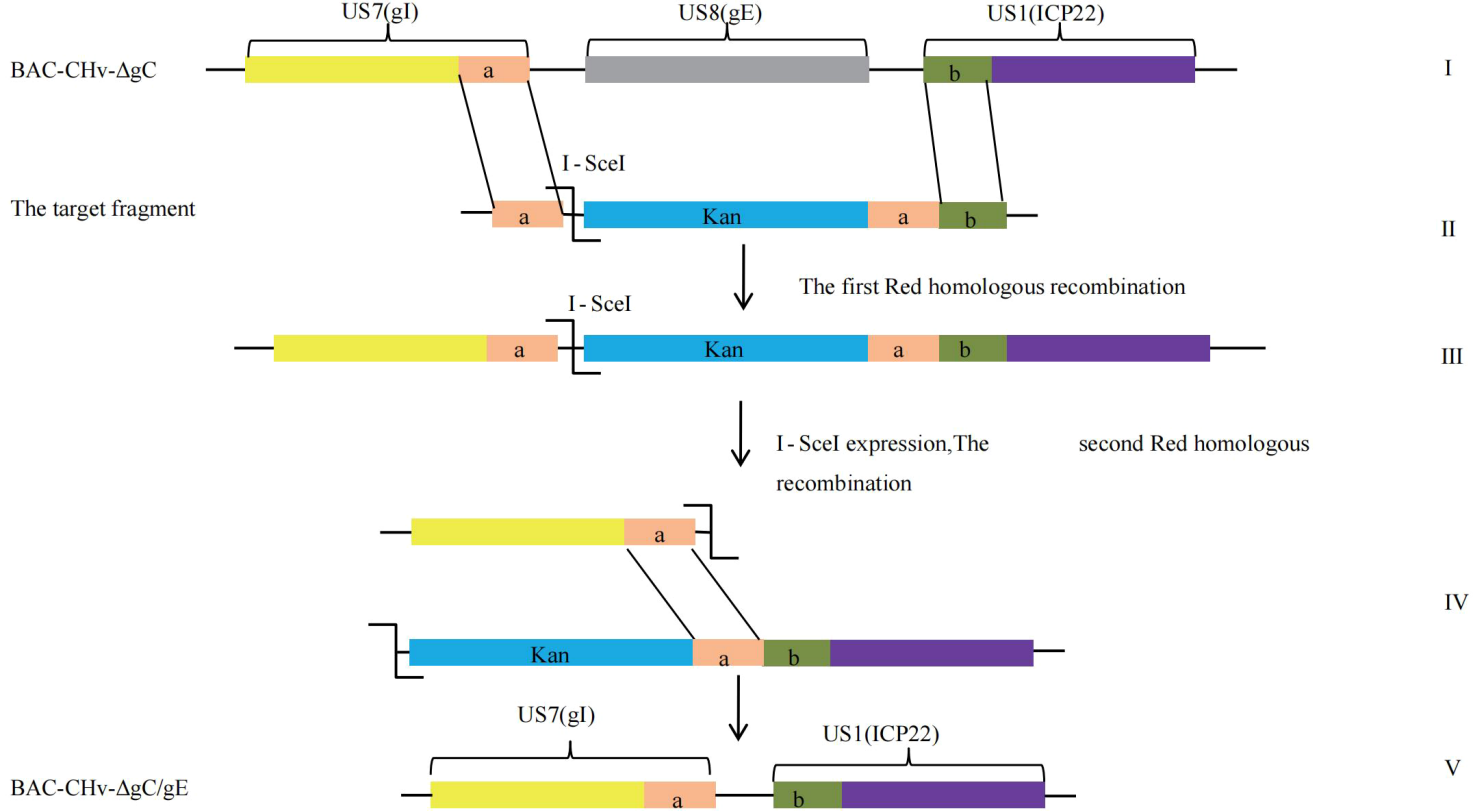
Homologous recombination diagram. Schematic diagram of constructing CHv-ΔgC/gE using Red recombination system. "a" and "b" represent the terminal sequences of the Us7 gene and the Us1 gene, respectively.

**Figure 2:**
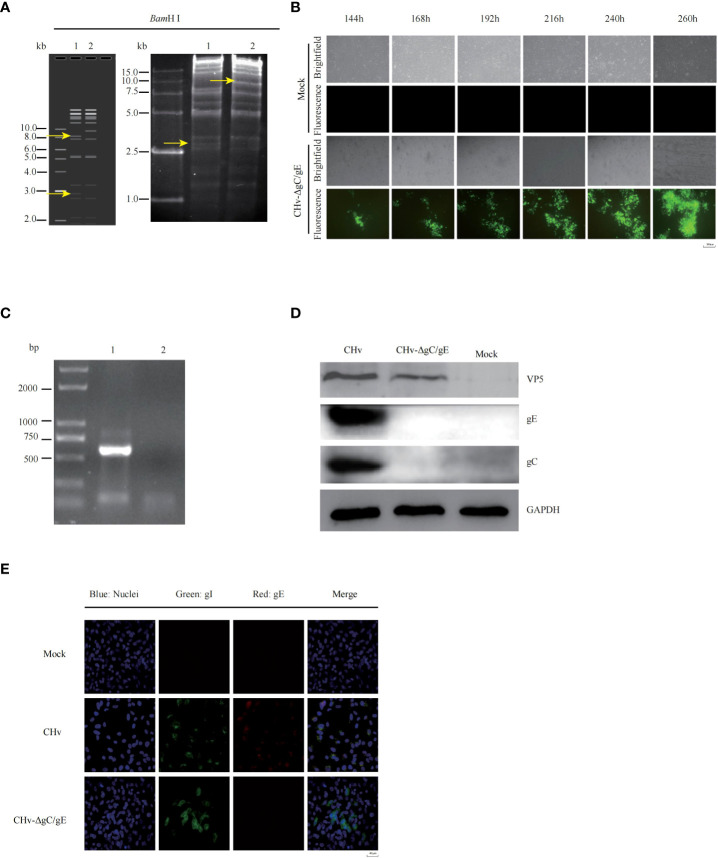
Generation and identification of recombinant CHv with deletion of gC/gE genes. **(A)** Predicted and actual RFLP analysis of CHv and CHv-ΔgC/gE infectious clones. The obtained DNA was digested with BamH1 and electrophoresed in a 0.7% agarose gel. (line 1:BAC-CHv, line 2: BAC-CHv-ΔgC/gE) **(B)** Transfection of the plasmids CHv-ΔgC/gE -GS1783 into DEFs resulted in numerous fluorescent spots and cytopathies, the mutant virus CHv-ΔgC/gE were rescued. **(C)** PCR analysis of the gE expression of CHv-ΔgC/gE. (line 1: CHv-ΔgC/gE, line 2: Mock) **(D)** DEFs were infected (MOI = 0.01) with CHv-ΔgC/gE or CHv. At 24 h post infection (hpi), the levels of gC and gE proteins were determined with western blotting. **(E)** DEFs were infected (MOI = 0.1) with CHv-ΔgC/gE, CHv. Expression of gE and gI proteins was detected by indirect immunofluorescence at 36 hpi.

In previous studies, it has been demonstrated that gE and gC are key genes of alphaherpesvirus immunosuppressive effect and virulence, and are essential for virulence. Here we constructed a DPV vaccine candidate in which the gE gene was deleted from the gC gene deletion strain CHv-ΔgC by two-step RED homologous recombination, and the mutant was named CHv-ΔgC/gE (**Figure 1**). RFLP analysis confirmed that the CHv-ΔgC/gE infectious clone was constructed correctly (**Figure 2A**). PCR products containing upstream and downstream regions of the gE sequence were amplified by gE identification primers and sequenced it (data not shown). Correctly sequenced plasmids were transfected into DEFs and green fluorescence was observed (**Figure 2B**). The rescued virus was identified by PCR using gE identification primers, the results showed that the size of the amplified fragment was consistent with the expectation of gE deletion. Combined with the analysis of the sequencing results, it showed that the virus had lacked gE (**Figure 2C**). To detect gE expression, DEFs were infected with CHv and CHv-ΔgC/gE, cells were lysed with RIPA buffer, and extracts were subjected to Western blot analysis. According to Western blot results, gC and gE proteins could be detected in CHv group, but not in CHv-ΔgC/gE group (**Figure 2D**). Indirect immunofluorescence (IFA) showed that no gE was detected when DEFs were infected with CHv-ΔgC/gE, but gE was detectable when infected with CHv (**Figure 2E**).

**Figure 3:**
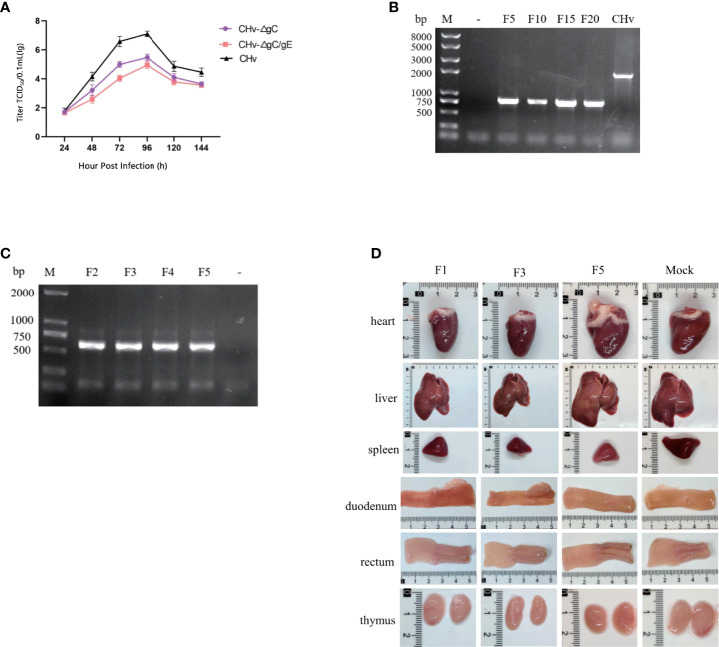
Stability of CHv-ΔgC/gE. **(A)** DEFs were infected with CHv-ΔgC/gE, CHv-ΔgC or CHv (MOI = 0.01). At indicated time points, cells and supernatants were harvested and virus titers were determined. **(B, C)** Levels of the gE genes were determined with PCR. **(B)** DEFs were infected with CHv-ΔgC/gE (10^6^ TCID_50_) and subjected to 20 passages (lines from left to right: Mock, CHv-ΔgC/gE F5, F10, F15, F20 and Parental virus). **(C, D)** ducklings were infected (10^6^ TCID_50_) with CHv-ΔgC/gE and subjected to 5 passages. **(C)** Identification of CHv-ΔgC/gE by PCR using gE identification primers (lines from left to right: CHv-ΔgC/gE F2, F3, F4, F5 and Mock). **(D)** Viscera observation of ducklings after challenge with CHv-ΔgC/gE (F1, F3, F5 and Mock).

Multi-step growth curve of CHv-ΔgC/gE strain showed that CHv-ΔgC/gE, CHv-ΔgC and CHv had similar proliferation curves, and the virus titers reached the highest 96 h after infecting cells. At 96 h, the mean titer of CHv-ΔgC/gE was 141-fold lower than that of CHv and 3-fold lower than that of CHv-ΔgC, indicating that the proliferative capacity of CHv-ΔgC/gE was further decreased after gE knockout (**Figure 3A**).

To confirm the genetic stability of CHv-ΔgC/gE, it was passaged 20 times on DEFs, and gE identification primers were used to detect gene deletion every 5 passages (**Figure 3B**). To determine the genetic stability *in vivo*, 20 14-day-old Peking ducks were divided into 2 groups, 10 in each group were injected intramuscularly with 10^6^ TCID_50_ CHv-ΔgC/gE and MEM in the right leg, respectively. After 7 days of infection, 5 ducks in each group were randomly slaughtered to observe tissue lesions, and the virus in the liver was used for passage and DNA extraction to detect the deletion of the gE gene (**Figure 3C**). During the *in vivo* passage, no obvious lesions were found in the organs of the slaughtered ducks (**Figure 3D**), and the remaining infected ducks were raised to 28 days of age. During this period, all infected ducks of each generation showed no obvious symptoms of duck plague and all survived, indicating that the virulence of the CHv-ΔgC/gE gene deletion strain remained stable during passage in ducks, and the virulence did not return to strong.

### Safety of CHv-ΔgC/gE in ducks

**Figure 4:**
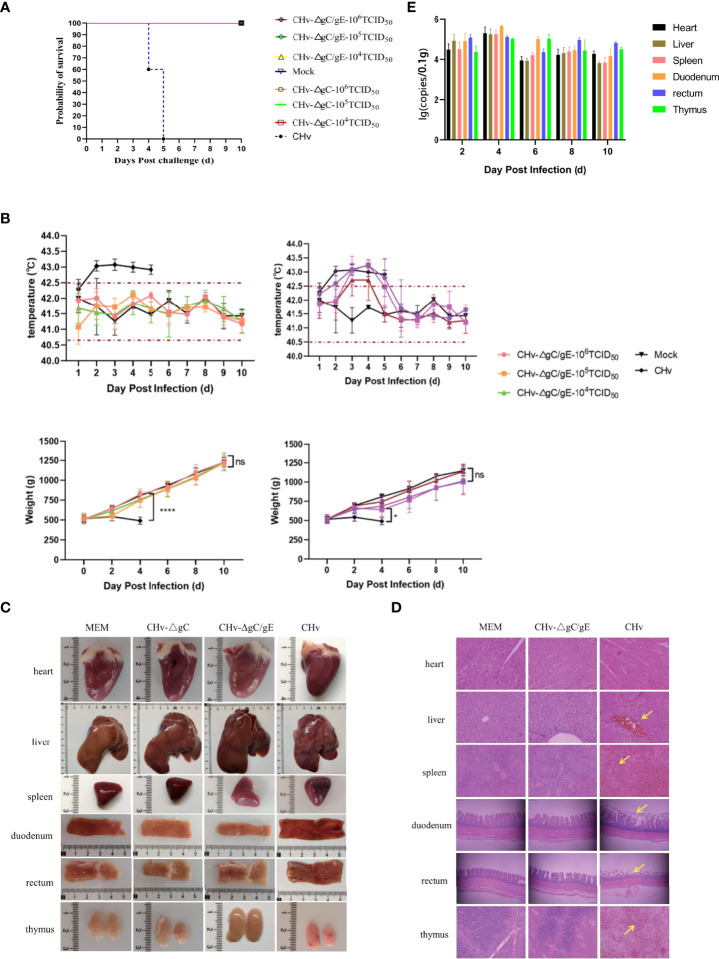
Pathogenicity of CHv-ΔgC/gE in ducklings. **(A)** Survival curves of ducklings after challenge with the indicated viral strains. **(B)** Daily body weight and temperature of all the ducklings after challenge with the indicated viral strains. **(C)** Postmortem examination of ducklings in each group at 5 dpi. **(D)** Histological analysis of heart, liver, spleen, duodenum, rectum and thymus from ducks injected with the indicated viral strains or MEM. Arrows indicate cellular infiltration or tissue disruption. (hematoxylin and eosin staining, 100× magnification). **(E)** Quantification of viral DNA loads in selected tissues with real-time PCR. Viral DNA copy numbers were determined with primers specific for UL30, as described above. *p<0.05; **p<0.01; ***p<0.001; ****p<0.0001; and ns, not significant.

The results of 14-day-old ducklings injected with CHv-ΔgC/gE, CHv-ΔgC, CHv and MEM showed that all the ducks in the CHv group died on the 5th day after infection, and all the ducks in the other groups survived (**Figure 4A**). The body temperature of ducks in MEM group and CHv-ΔgC/gE group fluctuated within the normal range (40.5-42.5°C) without fever. The ducks in the CHv-ΔgC group had different degrees of fever at the beginning of infection, but returned to normal body temperature after only 3 days, while the ducks infected with CHv virulent strain developed severe fever on the second day of infection, with body temperature exceeding 43°C, and the high temperature was maintained until all the ducks died on the 5th day (**Figure 4B**). During the observation period, the weight gain trend of the ducks inoculated with CHv-ΔgC/gE and CHv-ΔgC was consistent with that of the MEM group, showing a steady increase trend, while the weight of the ducks inoculated with CHv decreased until all died (**Figure 4B**).

To further confirm whether the CHv-ΔgC/gE strain is pathogenic to ducks, ducks were infected with CHv-ΔgC/gE, CHv-ΔgC and CHv. On the 5th day after infection, the duck was examined to observe tissue lesions, and the pathological section was made to observe the microscopic lesion. The necropsy showed that there was no abnormality in the organs of the ducks in the CHv-ΔgC/gE group and the negative control group; after infection with CHv-ΔgC, the thymus of the ducks showed slight hemorrhage, and other organs were not damaged; the CHv group, all organs except the heart had obvious abnormal conditions such as hemorrhage, congestion, necrosis and atrophy (**Figure 4C**). The organ samples of CHv-ΔgC/gE, CHv and MEM groups were collected to make microscopic pathological sections for observation. The results showed that there was no abnormal damage such as cell degeneration, inclusion bodies and inflammatory debris in the organs of the ducks in the MEM group and the CHv-ΔgC/gE group; In the CHv group, the ducks had no obvious lesions except the heart, other organs had obvious lesions. A large number of red blood cell infiltration in the central vein of the liver, diffuse hyperemia in the spleen, rupture of the villi membrane of the duodenum and the rectum, a large number of red blood cell infiltration in the thymus (**Figure 4D**).

### Distribution and viral load of CHv-ΔgC/gE in various tissues and organs of ducks

15 14-day-old ducks were infected with CHv-ΔgC/gE (10^6^TCID_50_), the organ samples of three ducks were collected randomly at 2, 4, 6, 8 and 10 dpi. The distribution of CHv-ΔgC/gE in ducks was observed by measuring virus copy number. The results showed that CHv-ΔgC/gE mainly invaded the intestinal tract of ducks, but the virus copy number remained at a low level. The duodenum and rectum had higher copy numbers than other organs at each detection time point, which was consistent with the natural characteristics of DPV infection (**Figure 4E**).

### The protective effect of CHv-ΔgC/gE on ducks

**Figure 5:**
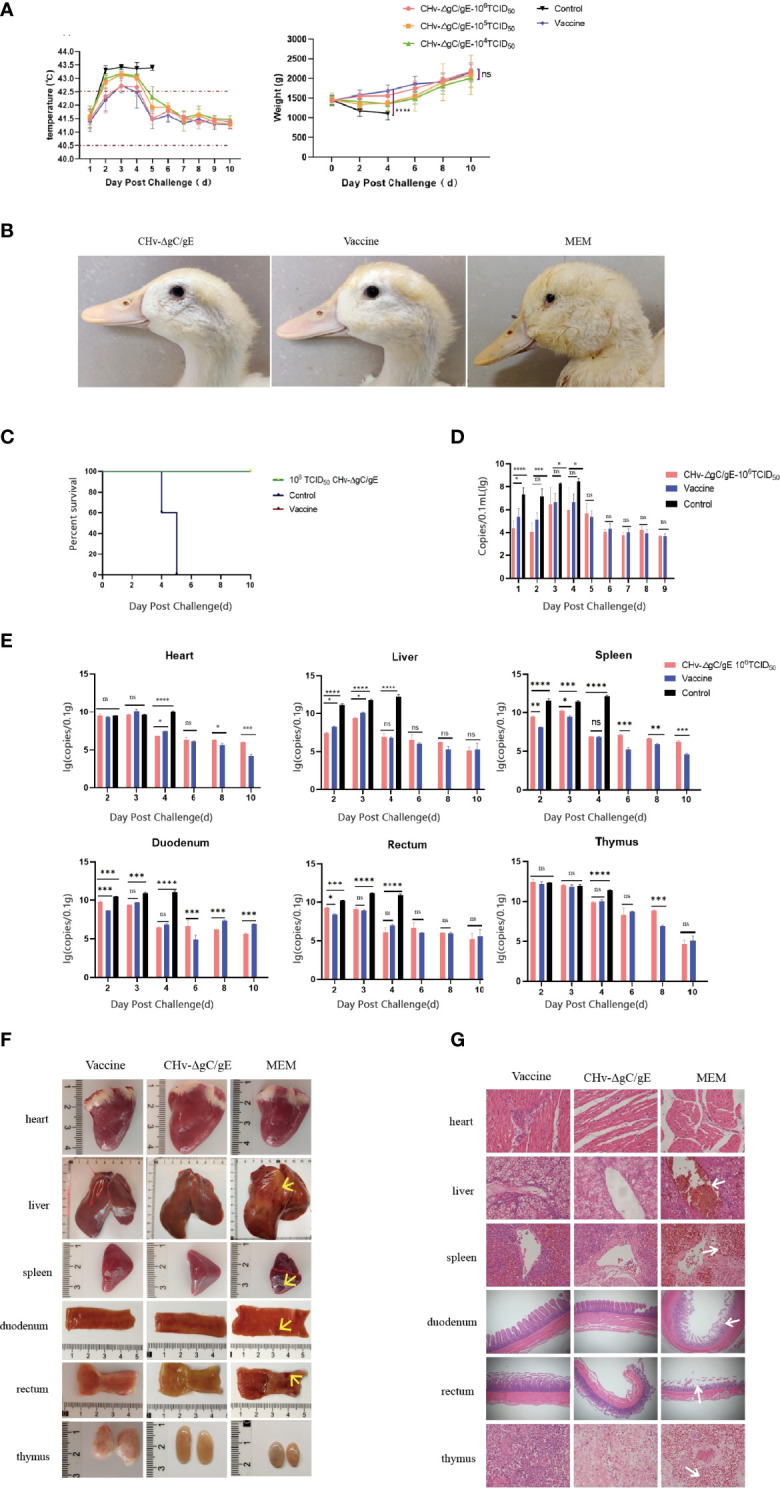
Protective efficacy of CHv-ΔgC/gE against lethal CHv challenge in ducklings. **(A)** Rectal temperatures and body weight changes of ducklings challenged after immunization. **(B)** Clinical symptoms of immunized ducklings after challenge with 100 LD_50_ CHv. **(C)** Survival curves of ducklings after challenge with 100 LD_50_ CHv. **(D)** Rectal viral excretion by ducklings after challenge with 100 LD_50_ CHv. **(E)** Quantification of viral DNA loads in heart, liver,spleen, duodenum, rectum and thymus tissues of the vaccinated duckings after challenge. **(F)** Postmortem examination of ducklings in each group at 6 dpi. There are obvious bleeding, congestion, ulcers and other lesions at the arrow. **(G)** Histological analysis of heart, liver,spleen, duodenum, rectum and thymus from the indicated groups after challenge with CHv-ΔgC/gE, Vaccine or MEM (control) (hematoxylin and eosin staining, 100× magnification). *p<0.05; **p<0.01; ***p<0.001; ****p<0.0001; and ns, not significant.

To determine whether the CHv-ΔgC/gE strain can protect ducks from lethal challenge with virulent CHv strains. 50 14-day-old ducks were randomly divided into 5 groups and inoculated with different doses of CHv-ΔgC/gE strain, AV1222 and MEM. Ducks were challenged with 100 LD_50_ CHv at 10 dpi. The MEM-vaccinated group started to have a fever at 2 dpc, with the temperature exceeded 43°C (**Figure 5A**). They also displayed typical duck plague symptoms, evident as lose weight, swollen head, teary eyes, lethargy and all died at 4~5 dpc (**Figures 5B, C**). However, ducks vaccinated with CHv-ΔgC/gE and Vaccine did not have any obvious clinical symptoms of duck plague, steadily increased body weight and all survived (**Figure 5A**). Viral excretion was measured by taking rectal swabs from each group within 9 dph. Ducks in the MEM inoculated group had significantly higher levels of shed virus before death than in the immunized group. The viral shedding in the CHv-ΔgC/gE group and the Vaccine group showed a trend of first increasing and then decreasing. The CHv-ΔgC/gE group was only significantly higher than that of the ducks in the Vaccine group at 1 dpc, and there was no significant difference at other times (p > 0.05) (**Figure 5D**).

In order to observe the protective effect of CHv-ΔgC/gE on duck organs. 54 14-day-old ducks were randomly divided into 3 groups and inoculated with CHv-ΔgC/gE, Vaccine and MEM, respectively. Ducks were challenged with 100 LD_50_ CHv 10 days after immunization, and the organs of three ducks were randomly collected from each group at 2, 3, 4, 6, 8, and 10 dpc. The copy number of virulent CHv in organs was detected based on gC gene. The results showed that ducks immunized with CHv-ΔgC/gE could effectively clear the virulent CHv in their bodies, and the amount of virulent virus was significantly reduced. However, the copy number of CHv in unimmunized ducks remained at a high level until death after challenge (**Figure 5E**). At 6 dpc, the necropsy results of the organs showed that no obvious lesions were found in the organs of the ducks in the CHv-ΔgC/gE group and the Vaccine group; the organs of the dead ducks in the MEM group had different degrees of hemorrhage and necrosis, atrophy and other lesions (**Figure 5F**). Microscopic results showed that no obvious lesions were found in the organs of the ducks in the CHv-ΔgC/gE group and the vaccine group, while the organs of the ducks in the control group showed pathological damage. Hepatic central venous congestion, spleen cell necrosis with extensive hemorrhage, intestinal villus rupture and intestinal wall thinning in duodenum and rectum, extensive necrosis of thymic tissue with obvious congestion and unclear cell boundaries (**Figure 5G**).

**Figure 6:**
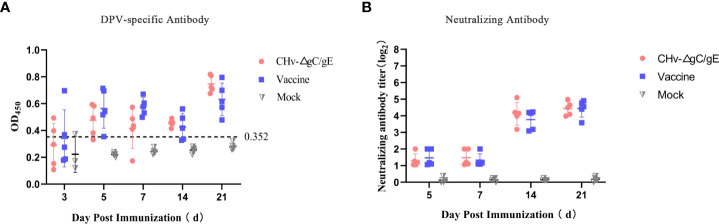
Production of CHv-specific antibodies in virus-inoculated ducklings. **(A)** The UL55-specific antibodies in the different groups at the indicated time points. The ELISA values for serum samples are given as OD450 and were deemed positive when OD_450_ > 0.352. **(B)** Serum neutralizing antibodies (NAbs) against the CHv in the indicated groups at 21 dpi.

To determine the immunogenicity of CHv-ΔgC/gE, 30 28-day-old Peking ducks were randomly divided into 3 groups, each group was injected with 10^6^ TCID_50_ CHv-ΔgC/gE, Vaccine and MEM in the right leg intramuscularly, and then collected serum samples at 1, 3, 5, 7, and 21 dpi. The results showed that the levels of DPV-UL55-specific antibodies and NAbs against DPV gradually increased in both vaccinated groups and there was no significant difference between the two groups. During the whole experiment, DPV-UL55-specific antibodies and anti-DPV NAbs were not detected in the MEM group (**Figures 6A, B**).

## Discussion

At present, the prevention of duck plague is the main measure to deal with duck plague. As early as the 1960s, domestic and foreign scholar have developed duck plague attenuated vaccines and inactivated vaccines to prevent and treat this disease (9). In practical production, duck embryonic attenuated vaccine and chicken embryo fibroblast vaccine are mainly used to control duck plague (14, 28, 29). Previous studies have shown that the deletion of viral genes or gene functional domains can reduce the virulence of herpes virus, have no pathogenicity or weaken pathogenicity to natural hosts, and can achieve protective effects, which can be used for further genetic engineering vaccine research (23, 30, 31). In this study, based on the constructed CHv-ΔgC single-gene deletion strain, the gE gene was deleted using the BAC gene editing system (24), and an attenuated CHv-ΔgC/gE strain with double gene deletion was obtained. Its genetic stability, pathogenicity to ducks and immunogenicity were evaluated in 14-day-old ducklings. During the passage of CHv-ΔgC/gE *in vitro* and *in vivo*, it has a high genetic stability and a low level of virulence. All the ducklings inoculated with high dose of CHv-ΔgC/gE did not show any clinical symptoms and no serious pathological damage to their organs. Importantly, after challenge with 100 LD_50_ CHv, the CHv-ΔgC/gE-vaccinated ducklings showed a significant reduction in organ viral load and shedding. These results suggest that CHv-ΔgC/gE is low toxicity and can effectively control DPV infection.

After deletion of *gC* and *gE*, the ability of CHv-ΔgC/gE strain to replicate and proliferate in ducklings is reduced, and it is not pathogenic to ducklings, indicating that *gC* and *gE* are important virulence genes of DPV. Studies have found that the deletion of part of the functional domain of gE will lead to the reduction of a large number of virion packaging, and the nucleocapsid cannot complete the secondary envelope coating, so that complete virions cannot be formed, which significantly inhibits the release of the virus (32). After mutating amino acids 208-236 of gE, gE and gI were unable to form heterodimers, which affected the distribution on the infected cell membrane and the spread of virions between cells (33). HSV cannot transmit normally from neurons to epithelial cells after mutation of amino acid 277 of HSV gE (22), after gE is deleted, the virus reversion from epithelial cells to neurons is also defective (21). The plaque area formed by CHv-ΔgE-infected cells was only 41.4% of that of the wild strain (24). The pathogenicity of the virus was significantly reduced after the deletion of the extracellular or intracellular domains of *gE*, CHv-gEΔET has a good protective effect on ducklings (23). Compared with CHv-gEΔET, the proliferation ability of CHv-ΔgC/gE constructed in this study was further reduced in DEFs, which indicated that CHv-ΔgC/gE was safer for ducklings. gC can combine with glycosaminoglycans to promote the adsorption and infection of the virus (34–36), combine with blood factors to promote the spread and replication of the virus in the body (37–39), gC can also inhibit complement-mediated neutralization by binding to complement C3b (40–42). Virus adsorption, *in vivo* dissemination and immune evasion mechanisms were affected after gC deletion. In conclusion, gC/gE is involved in the packaging, cell-to-cell transmission, and inter-neuronal transmission of virions, which may explain the reduction of virus virulence and the reduction of virus copy number in ducklings after the deletion of gC/gE.

In the study of DPV as a vector, the vaccine strain C-KCE has been proved to be an excellent vector, in which the insertion of foreign genes into the gE and gI positions of the vaccine strain is a common way (43–46). In this study, CHv-ΔgC/gE has no obvious pathogenicity to ducklings after immunization and can stimulate the body to produce antibodies for the same time as commercial vaccines, achieving the same protective effect as commercial vaccines, indicating that CHv-ΔgC/gE may also be an excellent carrier. CHv-ΔgC/gE has stable genetic characteristics, and can be produced in a cheaper and larger scale than traditional chicken embryo passaging live attenuated vaccines. In production practice, traditional live attenuated vaccines cannot distinguish naturally infected animals from immunized animals, while CHv-ΔgC/gE can be used as a marker vaccine to distinguish naturally infected animals from immunized animals according to the absence of gC/gE.

In conclusion, in this study, we constructed the recombinant virus CHv-ΔgC/gE through the BAC platform. The recombinant virus can induce ducks to produce DPV-UL55-specific antibodies and NAbs against DPV, is non-pathogenic to ducks, and can provide protection against DPV virulent lethal attack. Due to the deletion of gC and gE genes, CHv-ΔgC/gE can also be used as a marker vaccine to distinguish naturally infected animals from immunized animals. In addition, the good safety of CHv-ΔgC/gE strain can also be used as a carrier for combined vaccines.

## Data availability statement

The original contributions presented in the study are included in the article/supplementary materials. Further inquiries can be directed to the corresponding author.

## Ethics statement

All animal experiments were conducted in accordance with approved guidelines. 1-day-old Peking ducks were purchased from a farm operated by Sichuan Agricultural University (Sichuan, China). All experimental ducks did not contain DPV and were negative for DPV antibodies. All the ducks were housed in the animal facility at Sichuan Agricultural University, Chengdu, China. The study was approved by the Committee of Experiment Operational Guidelines and Animal Welfare of Sichuan Agricultural University (approved permit number XF2014-18).

## Author contributions

PR and AC contributed to the conception of the study. XF and PR performed the experiment. PR and MW performed the data analyses and wrote the manuscript. WZ, YW, QY, BT, XO, DS, SZ, SM contributed significantly to analysis and manuscript preparation. DZ, RJ, SC, ML, X-XZ, JH, QG helped perform the analysis with constructive discussions. All authors contributed to the article and approved the submitted version.

## Funding

This work was supported by National Natural Science Foundation of China (32072894),the China Agricultural Research System of MOF and MARA and Sichuan Veterinary Medicine and Drug Innovation Group of China Agricultural Research System (SCCXTD-2020-18).

## Conflict of interest

Author WZ is employed by Sinopharm Yangzhou Vac Biological Engineering Co. Ltd.

The remaining authors declare that the research was conducted in the absence of any commercial or financial relationships that could be construed as a potential conflict of interest.

## Publisher’s note

All claims expressed in this article are solely those of the authors and do not necessarily represent those of their affiliated organizations, or those of the publisher, the editors and the reviewers. Any product that may be evaluated in this article, or claim that may be made by its manufacturer, is not guaranteed or endorsed by the publisher.
